# Perinatal Asphyxia in Rat Alters Expression of Novel Schizophrenia Risk Genes

**DOI:** 10.3389/fnmol.2017.00341

**Published:** 2017-10-27

**Authors:** Alessandra Paparelli, Keiko Iwata, Tomoyasu Wakuda, Conrad Iyegbe, Robin M. Murray, Nori Takei

**Affiliations:** ^1^Department of Psychosis Studies, Institute of Psychiatry, King’s College London, London, United Kingdom; ^2^Research Center for Child Mental Development, University of Fukui, Fukui, Japan; ^3^Department of Biology, University of Padova, Padova, Italy; ^4^Department of Psychiatry, Hamamatsu University School of Medicine, Hamamatsu, Japan; ^5^Division of Neuropsychological Development and Health Sciences, United Graduate School of Child Development, Osaka University, Kanazawa University, Hamamatsu University School of Medicine, Chiba University and University of Fukui, Hamamatsu, Japan; ^6^Research Center for Child Mental Development, Hamamatsu University School of Medicine, Hamamatsu, Japan

**Keywords:** asphyxia, cesarean section, perinatal, schizophrenia, risk genes, animal model

## Abstract

Epidemiological studies suggest that obstetric complications, particularly those related to hypoxia during labor and delivery, are a risk factor for development of schizophrenia. The impact of perinatal asphyxia on postnatal life has been studied in a rodent model of global hypoxia, which is accompanied by cesarean section birth. This asphyxia model shows several behavioral, pharmacological, neurochemical, and neuroanatomical abnormalities in adulthood that have relevance to schizophrenia. Further, it is suggested that schizophrenia has a strong genetic component, and indeed novel candidate genes were recently identified by a genome-wide association study. Here, we examined alteration in the novel schizophrenia risk genes, *CNNM2, CSMD1*, and *MMP16* in the brains of rats undergoing cesarean section with or without global hypoxia. The brain regions studied were the prefrontal cortex, striatum, and hippocampus, which are all relevant to schizophrenia. Risk gene expression was measured at three time periods: neonatal, adolescence, and adulthood. We also performed an *in vitro* analysis to determine involvement of these genes in CNS maturation during differentiation of human neuronal and glial cell lines. *Cnnm2* expression was altered in the brains of asphyxia model rats. However, *Csmd1* and *Mmp16* showed altered expression by exposure to cesarean section only. These findings suggest that altered expression of these risk genes via asphyxia and cesarean section may be associated, albeit through distinct pathways, with the pathobiology of schizophrenia.

## Introduction

Schizophrenia is a mental disorder with an often chronic course, presenting with various symptoms including delusions, hallucinations, and impaired cognition. Multiple risk factors incorporating genetic susceptibility are associated with development of schizophrenia, indicating the underlying complexity of this debilitating condition. Epidemiological studies suggest that among many risk factors, obstetric complications, particularly those related to hypoxia during labor and delivery, are factors for increasing risk of schizophrenia (Dalman et al., 1999; Geddes et al., 1999; Zornberg et al., 2000; Cannon et al., 2008). Moreover, another line of investigation suggests a strong genetic component to schizophrenia. This is exemplified by a recent Genome-Wide Association Study (GWAS) that identified a number of genetic elements that predispose to schizophrenia (Gershon et al., 2011; Schizophrenia Psychiatric Genome-Wide Association Study [GWAS] Consortium, 2011; Aberg et al., 2013; Cross-Disorder Group of the Psychiatric Genomics Consortium, 2013; Ripke et al., 2013). On top of these separate viewpoints (i.e., environmental risk vs. genes), researchers highlight the likelihood of interplay between environmental risk and genes on predisposition to complex diseases such as schizophrenia (Ibi and Gonzalez-Maeso, 2015).

The impact of perinatal asphyxia on postnatal life has been studied using a rodent model of global hypoxia during cesarean section birth, known as the asphyxia model. There are several types of asphyxia models according to the time of asphyxia exposure. One model which employed 15 (±1) min asphyxia in the perinatal period has demonstrated several behavioral, pharmacological, neurochemical, and neuroanatomical abnormalities in adulthood, which are relevant to schizophrenia. For instance, the model has shown increased spontaneous locomotor activity and hypersensitivity to injection of apomorphine, amphetamine, and cocaine (Bjelke et al., 1991; Wakuda et al., 2008; Galeano et al., 2013). The same model has provided some evidence of impaired prepulse inhibition, implicated as a proxy measure for dysfunctional information processing underlying symptoms of schizophrenia (Fendt et al., 2008; Laplante et al., 2012). In addition, other studies that used the model have displayed a significant increase in catechol-*O*-methyltransferase (COMT) mRNA expression (Wakuda et al., 2015), and an increase in dopamine (DA) transporter density (Brake et al., 2000; El-Khodor and Boksa, 2000). In line with these findings, increased number of dopaminergic neurons has also been reported in the asphyxic rat brain (Bjelke et al., 1991). On the other hand, the other model which employed longer asphyxia (19–20 min) in the perinatal period has impairment in the GABAergic system in the striatum (Str) (Capani et al., 2009) and shown abnormal habituation memory in adulthood, which relevant to neurodevelopmental disorders (Saraceno et al., 2016). In this study, we placed importance on dopaminergic abnormalities in the 15 (±1) min asphyxia models. Because the abnormalities were in accordance with well-established findings in schizophrenia patients (Mohr and Ettinger, 2014). Therefore, we used the 15 min asphyxia rat as an animal model of schizophrenia. It has also been reported that cesarean section itself is associated with schizophrenia (Byrne et al., 2000), but so far this finding is inconsistent (Cannon et al., 2002). In rats, cesarean section (hereafter “C-section”) birth is sufficient to produce some abnormalities in the dopaminergic system, including increased spontaneous locomotor activity, hypersensitivity to amphetamine injection, and an increase in DA transporter density (Boksa and El-Khodor, 2003). Therefore, in this study, we also considered C-section as a potential exogenous insult.

As outlined above, previous asphyxia models have focused on the dopaminergic system. However, the process of asphyxia may also compromise other neural systems. Accordingly, we have previously found that expression of neuregulin 1 (*NRG1*), a schizophrenia risk gene (DeRosse et al., 2012), is significantly decreased in asphyxia-induced rats (Wakuda et al., 2015). The protein encoded by *NRG1* plays a role in regulation of synaptic plasticity and neurotransmission (Mei and Xiong, 2008). Thus, these studies suggest that a process induced by asphyxia during the perinatal period may involve alterations in not only the dopaminergic but also wider neural regulatory systems, and further, that these alterations may be mediated by genes.

Recently, novel five schizophrenia loci were identified by the Schizophrenia Psychiatric Genome-Wide Association Study [GWAS] Consortium (2011). This study provided high quality genetic data using substantial sample sizes and an optimal experimental design (Schizophrenia Psychiatric Genome-Wide Association Study [GWAS] Consortium 2011). Therefore, we determined if these novel schizophrenia risk genes show altered expression in asphyxia-induced rats. Among these five novel schizophrenia risk genes, we focused on *Cnnm2, Csmd1*, and *Mmp16* in this study. Because, risk variants of *CNNM2, CSMD1*, and *MMP16* are suggested to be involved in one of symptoms of schizophrenia, cognitive impairment (Koiliari et al., 2014; Rose et al., 2014; Morton et al., 2017). We measured gene expression at three periods: neonatal (postnatal day 1, P1), adolescence (5-week-old, 5W), and adulthood (12-week-old, 12W). These time frames are generally used for animal models of schizophrenia and correspond to the timing of asphyxia and/or C-section event, onset, and the chronic stage of schizophrenia in humans (Beninger et al., 2002; Baharnoori et al., 2009; Fatemi et al., 2009). We analyzed expression levels of schizophrenia risk genes in rat brain tissue, specifically, the prefrontal cortex (Pfc), Str, and hippocampus (Hip), which are suggested to be critically involved in schizophrenia (Bolkan et al., 2016). Little is known about whether expressions of *Cnnm2, Csmd1*, and *Mmp16* are involved in maturation of the central nervous system (CNS). Hence, we also examined expressions of the novel schizophrenia risk genes during the course of differentiation and maturation of neural and glial cell lines to confirm involvement of the genes (Schizophrenia Psychiatric Genome-Wide Association Study [GWAS] Consortium, 2011) in CNS maturation.

## Materials and Methods

### Animals and Induction of Perinatal Asphyxia

All animal experiments were performed in accordance with the Guide for Animal Experimentation at the Hamamatsu University School of Medicine. Intrauterine anoxia was induced in rats delivered by C-section according to a previously described method (Wakuda et al., 2008, 2015). Pregnant female Sprague-Dawley rats (Japan SLC, Hamamatsu, Japan) within the last day of gestation were anesthetised by diethyl ether, and hysterectomised. The uterus, including fetuses, was placed in a water bath at 37°C to induce 15 min of asphyxia, which is associated with 100% survival. After delivery, the umbilical cord was ligated, and the pups left to recover on a heating pad for at least 40 min. Rats that had delivered normally were used as surrogate mothers. Each surrogate mother received four vaginally delivered pups from another surrogate mother, four C-section-delivered, and four asphyxia-exposed pups. One day after birth (P1), brain tissue was collected from anesthetised male rats. Other male rats were housed three per cage in a temperature- and humidity-controlled colony room, maintained on a 12-h light/dark cycle (07:00 to 19:00 h light on) and with food and water provided *ad libitum*, and brain tissue was collected under anesthesia at 5 and 12 weeks after birth. The animals were divided into three groups based on their delivery: vaginal delivery (V group: *n* = 7), C-section (C group: *n* = 6), or C-section with 15 min of perinatal asphyxia (A group: *n* = 8). The Pfc, Str, and Hip were dissected on ice and used for the quantitative real-time reverse-transcription-polymerase chain reaction (qRT-PCR) analysis. The brain regions were defined according to the atlas of Paxinos and Watson (Wakuda et al., 2015).

### Cell Culture and Neuronal and Glial Differentiation

The SK-N-SH cell line was cultured in α-MEM (Nacalai Tesque, Kyoto, Japan) containing 10% (v/v) FBS (Life Technologies, Tokyo, Japan), penicillin (100 U/ml), and streptomycin (100 μg/ml) at 37°C in 5% CO_2_. The medium was changed every 2–3 days. The MO3.13 cell line was cultured in DMEM (Sigma–Aldrich) containing 10% (v/v) FBS (Life Technologies), penicillin (100 U/ml), and streptomycin (100 μg/ml) at 37°C in 5% CO_2_. Medium was changed every 2–3 days. To induce differentiations, SK-N-SH cells were treated with 10 μM ATRA (Maris and Matthay, 1999; Wainwright et al., 2001) (Sigma–Aldrich) and MO3.13 cells were treated with 100 nM PMA (McLaurin et al., 1995) (Sigma–Aldrich). DMSO (0.1%; Sigma–Aldrich) was used as the negative control for both differentiation inductions.

### RNA Isolation and Quantitative Real-Time Reverse-Transcription-Polymerase Chain Reaction (qRT-PCR)

Total RNA was isolated from brain tissue (including Pfc, Str, and Hip) and cell lines using TRIZOL Reagent (Invitrogen, Carlsbad, CA, United States). Total RNA was transcribed to cDNA using the SuperScript III First-Strand Synthesis System (Invitrogen). qRT-PCR was performed using SYBR Green (Qiagen, Hilden, Germany). Relative quantification of the rat genes: ancient conserved domain-containing protein 2 (Cnnm2), CUB and Sushi multiple domains 1 (Csmd1), and matrix metallopeptidase 16 (Mmp16); and the human genes: neuron-specific enolase (NSE), myelin basic protein (MBP), CNNM2, CSMD1, and MMP16 were determined using the delta-delta C_T_ method (Bookout and Mangelsdorf, 2003). The constitutive gene, Gapdh/GAPDH, was used as the internal control. mRNA levels were expressed as fold change relative to the V group. The primer sequences for qRT-PCR were as follows: Cnnm2 sense, 5′-TTGTCAGCAGGACAGAGGTG-3′; antisense, 5′-GTCGCTCCGACTGAGAGAGT-3′; Csmd1 sense, 5′-ATCATTACCAGGGCACCAG-3′; antisense, 5′-TTTTCATGGCCAGCATAGC-3′; Mmp16 sense, 5′-AGCTTTCGTCCACAAGGAAA-3′; antisense, 5′-CCTTGAGGATGGATCTTGGA-3′; Gapdh sense, 5′-GACATGCCGCCTGGAGAAAC-3′; antisense, 5′-AGCCCAGGATGCCCTTTAGT-3′; NSE sense, 5′-AGGCCAGATCAAGACTGGTG-3′; antisense, 5′-CACAGCACACTGGGATTACG-3′; MBP sense, 5′-ATGGCTAGACGCTGAAAACC-3′; antisense, 5′-AGGGGCAAGTGGGATTAAAG-3′; CNNM2 sense, 5′-GAAGCCATCCTGGACTTCAA-3′; antisense, 5′-CTCCCCTTCAAACACTGGAA-3′; CSMD1 sense, 5′-CTGCCATTCTGGTTCCTTTC-3′; antisense, 5′-CTGTTTTCATGCCCAGCATA-3′; MMP16 sense, 5′-AATCTCCTCAGGGAGCATTTGTA-3′; antisense, 5′-TCCAGGTTCTACCTTGAGTATCTG-3′; GAPDH sense, 5′-ATCAGCAATGCCTCCTGCAC-3′; and antisense, 5′-TGGCATGGACTGTGGTCATG-3′.

### Statistical Analysis

Quantitative gene expression in brain tissue was analyzed using a two-way multivariate analysis of variance (two-way MANOVA) and analysis of variance (ANOVA). In this model, the three separate brain areas (Pfc, Str, and Hip) in each rat were not independent and mutually correlated, and were therefore together treated as dependent variables to allow for applying two-way MANOVA. ANOVA followed by Tukey’s *post hoc* test was applied for analyzed gene expressions in individual group (V vs. A, C vs. A and V vs. A). For *in vitro* studies, two-tailed unpaired *t*-tests were used after no violation of the equal variance assumption was confirmed by *F*-test. All statistical analyses were set at a two-tailed α level of 0.05 for significance. The statistical analysis software, SPSS (version 12.0 J; SPSS, Inc., Chicago, IL, United States), was used for analyses.

## Results

### Gene Expression in Three Brain Regions at Different Time Periods Following Hypoxic Insult at Birth

Expression levels of these genes in Pfc, Str, and Hip in the V group (*n* = 7), C group (*n* = 6), and A group (*n* = 8) at three time points (P1, 5W, and 12W) were measured by qRT-PCR (Supplementary Table S1). First, we determined if gene expression levels were altered in C-section rats compared with the V group. Two-way MANOVA with a grouping factor (V and C groups) and time point (P1, 5W, and 12W) as the fixed factors, and gene expression in three brain regions (Pfc, Str, and Hip) as the dependent variables, was performed separately for each gene. This analysis identified no significant grouping effect (*F*_3,31_ = 0.54, *P* = 0.65) for *Cnnm2* expression, suggesting there is no difference in gene expression between the C and V groups. Thus, these two groups of rats (V and C groups) were amalgamated to yield a single control group in subsequent analyses. However, gene expression in the remaining two genes, *Csmd1* and *Mmp16*, was found to differ according to the presence or absence of C-section. There was a significant grouping effect (*F*_3,31_ = 17.94, *P* < 0.001 and *F*_3,31_ = 10.81, *P* < 0.001, respectively) for *Csmd1* and *Mmp16*. These results indicate a moderate effect of C-section on expression of these two genes.

**FIGURE 1:**
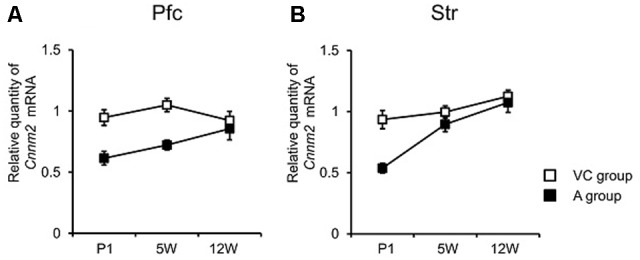
*Cnnm2* expression of different brain regions at different time periods following hypoxic insult at birth. quantitative real-time reverse-transcription-polymerase chain reaction (qRT-PCR) quantification of *Cnnm2* expression in prefrontal cortex (Pfc) **(A)** and striatum (Str) **(B)** at neonatal (postnatal day 1, P1) adolescence (5-week-old, 5W), and adulthood (12-week-old, 12W). Relative *Cnnm2* expression was obtained by normalizing to *Gapdh* from the same cDNA. Results are calculated as a ratio of V group expression. Error bars represent mean ± SEM (VC group, *n* = 13; A group, *n* = 8).

Following the MANOVA results comparing gene expression between the V and C groups, we next determined if *Cnnm2* gene expression differed between the A group and a combined control rat group (called the “VC group”) (**Table 1**). Two-way MANOVA revealed a significant grouping effect (*F*_3,55_ = 8.76, *P* < 0.001), and an overall difference in *Cnnm2* expression between asphyxia-induced and control rats, with *Cnnm2* expression significantly lower in the A group compared with the VC group. Subsequent univariate analyses found group differences present in Pfc (*P* < 0.001) and Str (*P* < 0.001), but not Hip (*P* = 0.18). In addition, a significant time × group interaction was detected in Str (*P* = 0.019), but not Pfc (*P* = 0.100) by two-way ANOVA. This difference in quantified *Cnnm2* mRNA was apparent immediately after asphyxia (i.e., at P1) in striatal tissue, but became less marked afterward (at 5W and 12W) (**Figure 1B**). In contrast, as indicated by the non-significant time × group interaction in Pfc, decreased gene expression in asphyxia rats was persistent across all three time points, although the difference was minimized at 12W (**Figure 1A**). In supporting of this, when we analyzed *Cnnm2* expression in individual group (V vs. A, C vs. A, and V vs. A) by ANOVA, group differences in Pfc (V vs. A, *P* < 0.001, Tukey’s *post hoc* test; C vs. A, *P* = 0.008, Tukey’s *post hoc* test) and Str (V vs. A, *P* = 0.018, Tukey’s *post hoc* test; C vs. A, *P* = 0.004, Tukey’s *post hoc* test) were also found (Supplementary Figures S1A,B).

**Table 1:** Cnnm2 in three brain regions at different time periods following hypoxic insult at birth.

Gene name	Brain region	VC group			A group		
		P1	5W	12W	P1	5W	12W
*Cnnm2*	Pfc	0.95 ± 0.06	1.05 ± 0.05	0.92 ± 0.07	0.61 ± 0.06	0.72 ± 0.04	0.86 ± 0.09
	Str	0.93 ± 0.05	1.00 ± 0.05	1.13 ± 0.05	0.54 ± 0.04	0.90 ± 0.06	1.07 ± 0.08
	Hip	0.88 ± 0.05	1.17 ± 0.08	0.92 ± 0.04	0.68 ± 0.05	1.09 ± 0.04	0.97 ± 0.09

*Quantitative real-time reverse-transcription-polymerase chain reaction (qRT-PCR) quantification of *Cnnm2*expression in prefrontal cortex (Pfc), striatum (Str), and hippocampus (Hip) at neonatal (postnatal day 1, P1), adolescence (5-week-old, 5W), and adulthood (12-week-old, 12W). Values are expressed as mean ± SEM.*

Initial analyses revealed that *Csmd1* and *Mmp16* gene expression differed between the C and V groups, that is, due to the presence or absence of C-section. This finding of differing gene expression in association with C-section led us to speculate that C-section itself could be considered to be an exogenous insult, and may play a role in altering brain expression of these two genes. Further, we found no additional influence of asphyxia on expression levels of either gene, when the A group (asphyxia + C-section) and C group (C-section only) were compared (*F*_3,34_ = 0.49, *P* = 0.69 and *F*_3,34_ = 0.678, *P* = 0.57; for *Csmd1* and *Mmp16* expression, respectively). Because there was no difference in gene expression between rats that had received a C-section, we combined the two rat groups (A and C groups) to yield one C-section group (CS group). Next, we compared gene expression levels between the V and CS groups (**Table 2**).

**Table 2:** Csmd1 and Mmp16 expression in three brain regions at different time periods following C-section at birth.

Gene name	Brain region	V group			CS group		
		P1	5W	12W	P1	5W	12W
*Csmd1*	Pfc	1.00 ± 0.08	1.00 ± 0.09	1.00 ± 0.15	2.46 ± 0.58	1.07 ± 0.04	0.86 ± 0.09
	Str	1.00 ± 0.03	1.00 ± 0.12	1.00 ± 0.08	1.55 ± 0.11	1.14 ± 0.08	1.34 ± 0.07
	Hip	1.00 ± 0.06	1.00 ± 0.05	1.00 ± 0.05	1.94 ± 0.24	1.19 ± 0.09	0.92 ± 0.08
*Mmp16*	Pfc	1.00 ± 0.06	1.00 ± 0.08	1.00 ± 0.17	2.70 ± 0.72	1.15 ± 0.11	0.95 ± 0.09
	Str	1.00 ± 0.07	1.00 ± 0.10	1.00 ± 0.04	1.69 ± 0.19	0.97 ± 0.06	1.16 ± 0.06
	Hip	1.00 ± 0.06	1.00 ± 0.05	1.00 ± 0.04	1.55 ± 0.27	1.13 ± 0.08	1.43 ± 0.45

*Quantitative real-time reverse-transcription-polymerase chain reaction quantification of *Cnnm2*expression in prefrontal cortex (Pfc), striatum (Str), and hippocampus (Hip) at neonatal (postnatal day 1, P1), adolescence (5-week-old, 5W), and adulthood (12-week-old, 12W). Values are expressed as mean ± SEM.*

**FIGURE 2:**
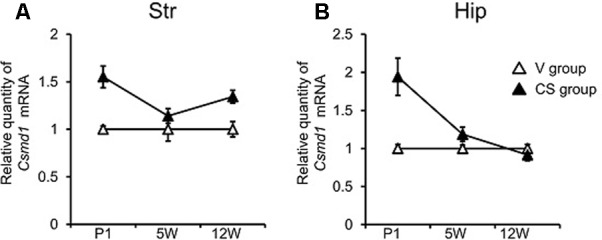
Csmd1 expression of different brain regions at different time periods following C-section at birth. qRT-PCR quantification of Csmd1 expression in Str **(A)** and hippocampus (Hip) **(B)** at neonatal (postnatal day 1, P1) adolescence (5-week-old, 5W), and adulthood (12-week-old, 12W). Relative Csmd1 expression was obtained by normalizing to Gapdh from the same cDNA. Results are expressed as a ratio of V group expression, resulting in a V group ratio of 1. Error bars represent mean ± SEM (V group, *n* = 7; CS group, *n* = 14).

As for *Csmd1*, two-way MANOVA revealed a significant grouping effect (*F*_3,55_ = 12.104, *P* < 0.001). Univariate analyses found group differences in Str (*P* < 0.001) and Hip (*P* = 0.011), but not Pfc (*P* = 0.085). Subsequent ANOVA showed a significant time × group interaction in Hip (*P* = 0.007), but not Str (*P* = 0.128). As implicated by the non-significant time × group interaction, increased *Csmd1* expression in the CS group in Str was relatively constant across all three time points, although the difference was somewhat minimized at 5W (**Figure 2A**). In Hip, elevated *Csmd1* expression was present at P1 (i.e., immediately after C-section), but became less marked afterward (i.e., at 5W and 1W) (**Figure 2B**). We also found group differences of *Csmd1* expression in individual group in Str (V vs. A, *P* = 0.002, Tukey’s *post hoc* test; V vs. C, *P* = 0.002, Tukey’s *post hoc* test) and Hip (V vs. C, *P* = 0.017, Tukey’s *post hoc* test) by ANOVA (Supplementary Figures S2A,B). There is no group difference between V and A in Hip. It may due to low statistical power because of small sample size.

**FIGURE 3:**
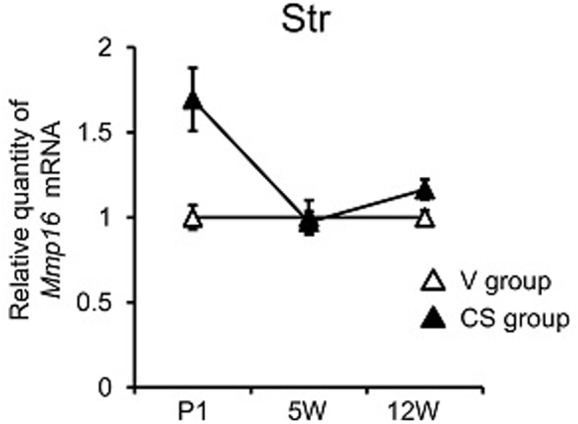
Mmp16 expression of different brain regions at different time periods following C-section at birth. qRT-PCR quantification of Mmp16 expression in Str at neonatal (postnatal day 1, P1) adolescence (5-week-old, 5W), and adulthood (12-week-old, 12W). Relative Mmp16 expression was obtained by normalizing to Gapdh from the same cDNA. Results are expressed as a ratio of V group expression, resulting in a V group ratio of 1. Error bars represent mean ± SEM (V group, *n* = 7; CS group, *n* = 14).

With regard to *Mmp16*, two-way MANOVA revealed a significant grouping effect (*F*_3,55_ = 4.60, *P* = 0.006). Subsequent univariate analyses indicated that the group difference was present in Str only (*P* = 0.009), while two-way ANOVA detected a significant time × group interaction in Str (*P* = 0.016). *Mmp16* expression in Str began at a higher level in the CS group than the V group at P1, but the difference became less marked afterward (**Figure 3**). We also found group difference of *Mmp16* expression in individual group in Str (V vs. C, *P* = 0.012, Tukey’s *post hoc* test) by ANOVA (Supplementary Figure S3). There is no group difference between V and A, may due to low statistical power because of small sample size.

### Altered Expression of Novel Schizophrenia Risk Genes during Differentiation of Neuronal and Glial Cell Lines

To investigate involvement of these genes in CNS development, we measured mRNA expression during neuronal differentiation and glial maturation in human cell lines. The human neuroblastoma cell line, SK-N-SH, was treated with ATRA (10 μM) to induce neuronal differentiation. After 72 h treatment, gene expression of the neuronal differentiation maker, NSE, was clearly increased (*P* < 0.001, two-tailed unpaired *t*-test) (Supplementary Figure S4A). Additionally, the human oligodendrocytic cell line, MO3.13, was treated with PMA (100 nM) to induce oligodendrocytic maturation. After 96 h treatment, gene expression of the oligodendrocytic differentiation maker, MBP, was substantively increased (*P* < 0.001, two-tailed unpaired *t*-test) (Supplementary Figure S4B).

**FIGURE 4:**
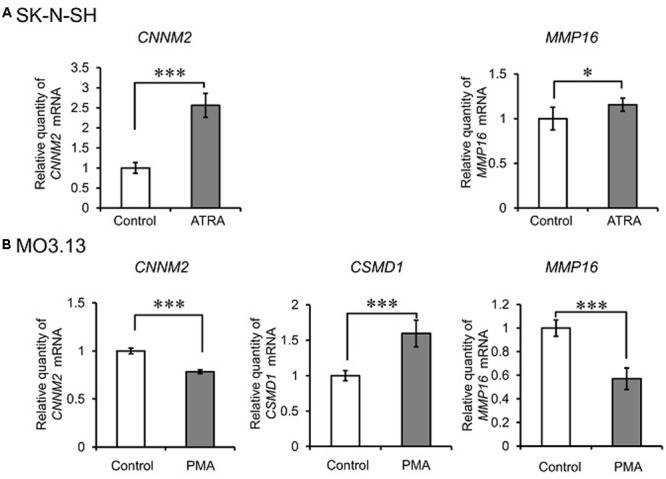
Alteration of expression of novel schizophrenia risk genes during differentiation of neuronal and glial cell lines. qRT-PCR quantification of CNNM2, CSMD1, and MMP16 expression during neuronal differentiation of SK-N-SH cells **(A)** and oligodendrocytic maturation of MO3.13 cells **(B)**. Relative CNNM2, CSMD1, and MMP16 expression were obtained by normalizing to GAPDH from the same cDNA. Results are expressed as a ratio of control (undifferentiated cells) expression, resulting in a control ratio of 1. Error bars represent mean ± SD (*n* = 3–4 per treatment). ^∗^*P* < 0.05, ^∗∗∗^*P* < 0.001 between control cells and cells treated with ATRA (for SK-N-SH cells) or PMA (for MO3.13 cells).

We then measured *CNNM2, CSMD1*, and *MMP16* expression during the differentiation processes. During neuronal differentiation, *CNNM2* and *MMP16* expression were significantly increased (*P* < 0.001 and *P* = 0.031, respectively) (**Figure 4A**). *CSMD1* was undetectable in both undifferentiated and differentiated SK-N-SH cells. During oligodendrocytic maturation, *CNNM2* and *MMP16* expression were significantly decreased (both *P* < 0.001), while *CSMD1* expression was significantly increased in MO3.13 cells (*P* < 0.001) (**Figure 4B**).

## Discussion

Here, we investigated the schizophrenia risk genes, *CNNM2, CSMD1*, and *MMP16* (Schizophrenia Psychiatric Genome-Wide Association Study [GWAS] Consortium, 2011), and identified alterations in gene expression in the asphyxia rat model for schizophrenia. In addition, we examined the influence of C-section on gene expression. Interestingly, we found that among the three genes examined, *Cnnm2* was related specifically to the asphyxia process, whereas the other two (*Csmd1* and *Mmp16*) were associated with C-section. *Cnnm2* expression was significantly downregulated in Pfc and Str under the influence of asphyxia. While such downregulation was observed only at the neonatal period in Str, it was sustained into adolescence in Pfc. In contrast, exposure to C-section was related to upregulated *Csmd1* and *Mmp16* expression. Moreover, altered *Csmd1* expression levels were conserved until adulthood in Str, but were transient in Hip. Furthermore, *Mmp16* expression was strikingly upregulated, but only immediately after C-section in Str. Using human cell lines, we provided some evidence indicative of potential involvement of *CNNM2, CSMD1*, and *MMP16* in CNS development.

### Effect of Asphyxia on *CNNM2* Expression

*CNNM2* is a member of the ancient conserved domain-containing protein family (Wang et al., 2003). Members of this protein family contain a sequence motif that is present in the cyclin box, specifically, a cyclic nucleotide-monophosphate (cNMP)-binding domain (Wang et al., 2003). The *CNNM2* gene has a ubiquitous expression pattern in humans (Wang et al., 2003), and is highly expressed in the brain^1^. However, little is known about expression levels of *CNNM2* in the brain of schizophrenia patients compared with control subjects.

To our knowledge, this is the first study to show that *Cnnm2* expression is downregulated in Pfc and Str in the asphyxia model. Notably, the reduction was maintained until adulthood in Pfc, but was restricted to the perinatal period in Str. It has been reported that schizophrenia patients with obstetric complications show worsened Pfc dysfunction, such as higher intensity of negative symptoms and worse performance on Wisconsin Card Sorting Test (WCST) measures, compared to those without complications (Borkowska and Rybakowski, 2002). This suggests that the event of asphyxia may affect CNS-related gene expression, and altered gene expression continues to play a role in disturbing brain function, resulting in formation of schizophrenia symptoms.

In our human cell line experiments, we found increased *CNNM2* expression during neuronal differentiation. Additionally, because our *in vitro* experiments show that mature neurons express *CNNM2* more than undifferentiated cells, and further, that *CNNM2* expression is reduced in asphyxia-induced rats, it is possible that mature neurons may be decreased, especially in the prefrontal region in the asphyxia model. Intriguingly, in line with our findings, it has been reported that a *CNNM2* variant (Schizophrenia Psychiatric Genome-Wide Association Study [GWAS] Consortium, 2011) is associated with gray matter morphological vulnerability of the bilateral inferior frontal gyri (Ohi et al., 2013).

A number of brain imaging studies have demonstrated decreased myelin integrity in schizophrenia (Martins-de-Souza, 2010). However, the maturation status of oligodendrocytes in the brain of schizophrenia patients is unknown. Of note in this context, is a study showing abnormal cell cycle re-entry in postmitotic oligodendrocytes in the brains of schizophrenia patients, with mitotically active oligodendrocytes functionally deficient in schizophrenia (Katsel et al., 2008). Intriguingly, it has been suggested that cell division increases the number of mitotically active oligodendrocytes (Katsel et al., 2008). In addition, mRNA levels of myelin-associated oligodendrocyte basic protein (MOBP), an oligodendrocyte-associated gene, are increased in Pfc white matter in schizophrenia patients (Mitkus et al., 2008). We found that reduced *Cnnm2* expression levels correlated with enhanced oligodendrocytic maturation in the human SK-N-SH cell line. Thus, our finding of reduced *Cnnm2* expression in the asphyxia model suggests that an accelerated maturational process may take place in oligodendrocytes from an early developmental stage, due to asphyxia exposure during the perinatal period, resulting in abnormal regulation of the oligodendrocyte cell cycle and an unusual maturation pattern. This scenario may be relevant to disturbed myelin integrity in schizophrenia.

CNNM2 has been proposed as a Mg^2+^ transporter in Xenopus oocytes (Goytain and Quamme, 2005), whereas human CNNM2 is a principal molecular factor of intracellular Mg^2+^ homeostasis but not a Mg^2+^ transporters *per se* (Sponder et al., 2016). Intriguingly, knockdown of *CNNM2* orthologs in zebrafish resulted in impaired brain development and reduced body Mg content (Arjona et al., 2014). Further study is needed to understand the underlying molecular mechanisms of CNNM2 in the brain development and the pathophysiology of schizophrenia.

Here, we failed to find any change in *Cnnm2* expression in Hip in the asphyxia model, despite numerous studies reporting various functional and structural hippocampal changes in the asphyxia model (Boksa, 2004). There is the possibility that hippocampal abnormalities in relation to asphyxia may be accounted for by genes other than the genes examined in this study.

### Effect of C-Section on CSMD1 and MMP16 Expression

Despite evidence showing that undergoing C-section at birth is itself associated with later development of schizophrenia (Byrne et al., 2000), there is inconsistency in the literature (Cannon et al., 2002). C-section delivery is also associated with other types of neurodevelopmental disorders, such as autism spectrum disorders and attention deficit hyperactivity disorder (Curran et al., 2015). We found that C-section was related to increased *Csmd1* and *Mmp16* expression.

*CSMD1* encodes the CUB and Sushi multiple domains 1 protein. CSMD1 protein is highly expressed in regions of neuronal differentiation and outgrowth, and remains high in the adult in areas of increased neuronal plasticity, such as the cerebral cortex and Hip (Kraus et al., 2006). It has been suggested that CSMD1 is an important regulator of complement activation and inflammation in the developing CNS, and also that it plays a role in growth cone function (Kraus et al., 2006). Recently, a *CSMD1* variant (Schizophrenia Psychiatric Genome-Wide Association Study [GWAS] Consortium, 2011) was associated with comparatively reduced cortical activation in the middle occipital gyrus and cuneus, a posterior brain region that supports maintenance processes during performance of a spatial working memory task, and affects general cognitive ability and executive function in healthy persons (Koiliari et al., 2014). In addition to this variant (Schizophrenia Psychiatric Genome-Wide Association Study [GWAS] Consortium, 2011), other variants of this gene have been associated with increased risk for schizophrenia (Havik et al., 2011; Sakamoto et al., 2016) and associated with illness severity at end point (Sakamoto et al., 2016).

This study is the first to show increased *Csmd1* expression in Str and Hip in relation to C-section. Of particular interest is the long-lasting change in striatal expression throughout adulthood. This is in accordance with the findings of long-term changes in dopaminergic parameters, such as tyrosine hydroxylase activity and DA transporter density in Str caused by C-section in the rat (El-Khodor and Boksa, 2003; Boksa and Zhang, 2008). Further, it has been reported that genetic variation of *CSMD1* (Schizophrenia Psychiatric Genome-Wide Association Study [GWAS] Consortium, 2011) plays a role in the ratio between DA and serotonin metabolites in CSF (Luykx et al., 2014). These studies suggest that the event of C-section may intervene in gene expression, particularly *CSMD1* expression in Str, and altered gene expression continues to play a role in disturbing function of the monoaminergic systems (in particular the dopaminergic system), resulting in psychotic symptoms. In human SK-N-SH cells, we found that *CSMD1* was involved in oligodendrocytic maturation. Along with this, the fact that *Csmd1* expression is upregulated in the C-section model suggests that C-section may exert a facilitatory effect on oligodendrocyte maturation. Further, a metabolic connection has been demonstrated between myelinating oligodendrocytes and axons (Funfschilling et al., 2012), and metabolite levels in fronto-striatal-thalamic white matter pathways differ in patients with schizophrenia compared with control subjects (Beasley et al., 2009). As mentioned above, it has been suggested that abnormal regulation of the oligodendrocyte cell cycle and an unusual maturation pattern may underlie schizophrenia (Katsel et al., 2008; Mitkus et al., 2008). It is possible that myelin integrity may be disturbed by unusual oligodendrocyte developmental processes in schizophrenia. These processes may be mediated by long-lasting changes in risk gene expression.

*MMP16* encodes for a matrix metalloproteinase family protein, which is involved in extracellular matrix breakdown in normal physiological processes such as embryonic development, reproduction, and tissue remodeling, as well as in physical disease processes (Andersson et al., 1996). Mmp16 mRNA is strongly expressed in the rat brain (Shofuda et al., 1997), and equally expressed in gray and white matter, suggesting possible roles for MMP16 in CNS development and/or function (Yoshiyama et al., 1998).

We found that C-section led to upregulated *Mmmp16* expression in Str, which was observed immediately after C-section. We also found that gene expression increased during neuronal differentiation and decreased during oligodendrocytic maturation in human cell lines. Gray and white matter abnormalities are present in schizophrenia at illness onset (Douaud et al., 2007). Therefore, it is possible that changes in *MMP16* expression early in life may compromise gray and white matter construction by accelerating neural differentiation and affecting oligodendrocyte maturation.

Molecular mechanisms which mediate the influence of CSMD1 and MMP16 on brain development and the pathobiology of schizophrenia remain unresolved. Further research is needed exploring these mechanisms.

In this study, we considered both asphyxia and C-section to equally play a role in predisposition to schizophrenia phenotypes. However, aetiological engagement of C-section might be weaker than asphyxia, because epidemiological studies of the former are inconsistent (Byrne et al., 2000; Cannon et al., 2002). Rat pups born by C-section show normal or slightly reduced oxygen partial pressure levels at birth and signs of mild respiratory distress during the 1st day of life (El-Khodor and Boksa, 1997; Vaillancourt et al., 1999; Berger et al., 2000). Thus, mild hypoxia (but not the level required for asphyxia) may be induced by C-section. Of note, human neonates born by C-section also suffer from increased occurrence of mild respiratory distress during the 1st days of life (Hales et al., 1993; van den Berg et al., 2001). However, since circumstances surrounding C-section in clinical practice may be complex, animal models may not wholly represent all aspects of C-section as occurs in humans. Indeed, Cannon et al. (2002) reported significant differences between schizophrenic and comparison subjects for emergency C-sections but not for elective C-sections.

Our model of asphyxia and C-section rats showed more salient changes in those gene expressions in Str relative to Pfc and Hip. These findings are in line with the view that Str is one of the most sensitive areas to asphyxia (Loidl et al., 1994; Fabian Loidl et al., 1997).

### Limitation

In this study, we examined multiple genes in three brain regions at three time points. There is a potential error in interpretation due to the nature of multiple testing. To attenuate this risk, we analyzed intergroup differences in three brain regions, instead of each brain region separately, by applying MANOVA. Based on the results, we proceeded to univariate analyses. Following Rothman’s suggestion (Rothman, 1990), we did not thoroughly adjust for multiple comparisons in the analysis, which may have led to eliminating any heuristic indication of results from this study. Nevertheless, there is a potential risk of false-positives, and further study is clearly warranted.

## Ethics Statement

The animal protocol used in this study was approved by an Institutional Animal Care and Use Committee at the Hamamatsu University School of Medicine. All animal experiments were performed in accordance with the Guide for Animal Experimentation at the Hamamatsu University School of Medicine.

## Author Contributions

AP, KI, RM, and NT designed the study. AP, KI, TW, and CI collected the data. AP, KI, RM, and NT analyzed and interpreted the data. KI and NT prepared the manuscript. All authors read and approved the final manuscript.

## Conflict of Interest Statement

The authors declare that the research was conducted in the absence of any commercial or financial relationships that could be construed as a potential conflict of interest.

## Footnotes

1 http://www.ebi.ac.uk/gxa/experiments/E-MTAB-513?geneQuery=ENSG00000148842
