# Supplementary figures and images for: Perinatal Asphyxia in Rat Alters Expression of Novel Schizophrenia Risk Genes
Source: Front Mol Neurosci. 2017 Oct 27;10:341. doi: 10.3389/fnmol.2017.00341 (PMC5663725; doi:10.3389/fnmol.2017.00341)

Supplemental figure 1

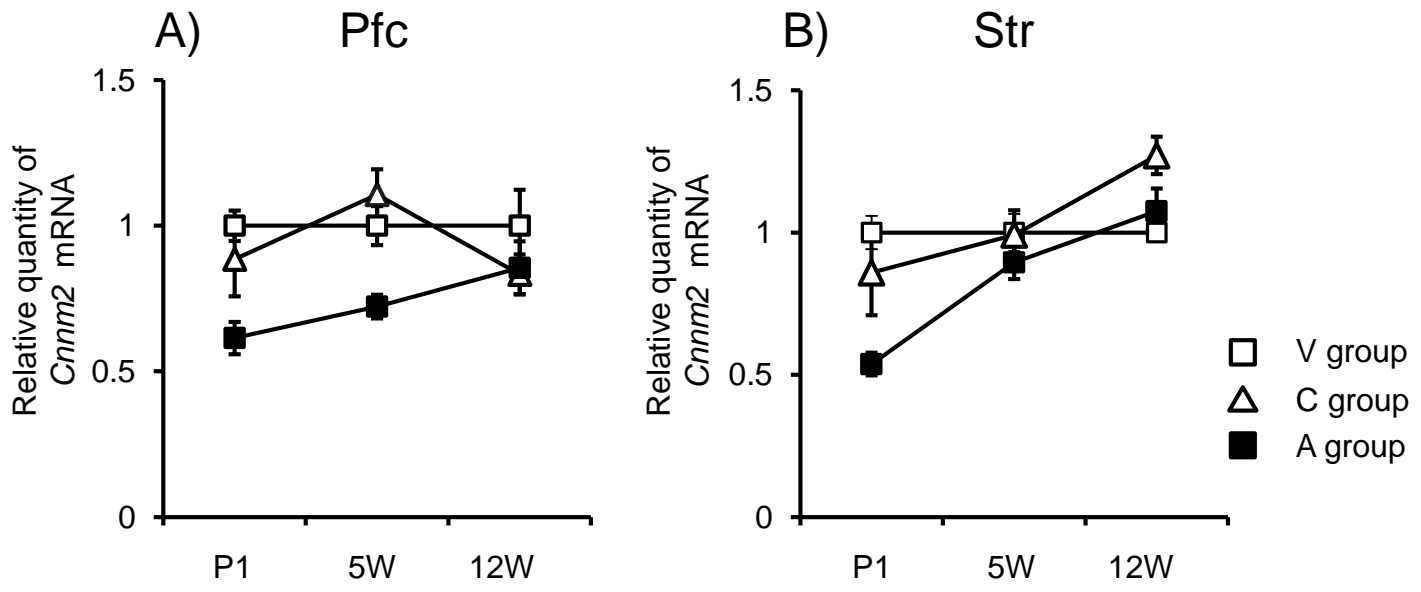

Supplement: Supplementary file 1 [file Presentation_1.PDF]

Supplemental figure 2

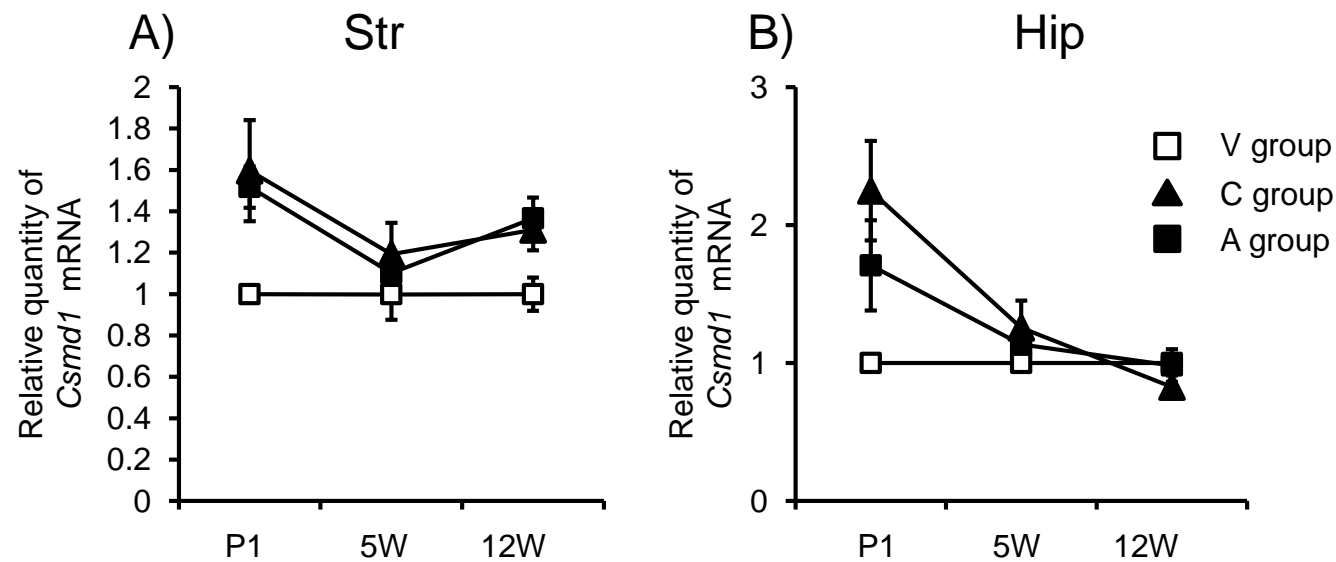

Supplement: Supplementary file 2 [file Presentation_2.PDF]

Supplemental figure 3

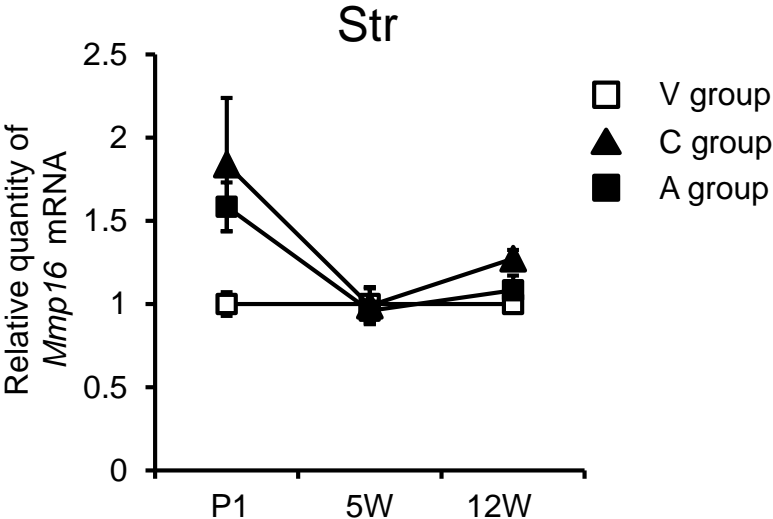

Supplement: Supplementary file 3 [file Presentation_3.PDF]

Supplemental figure 4

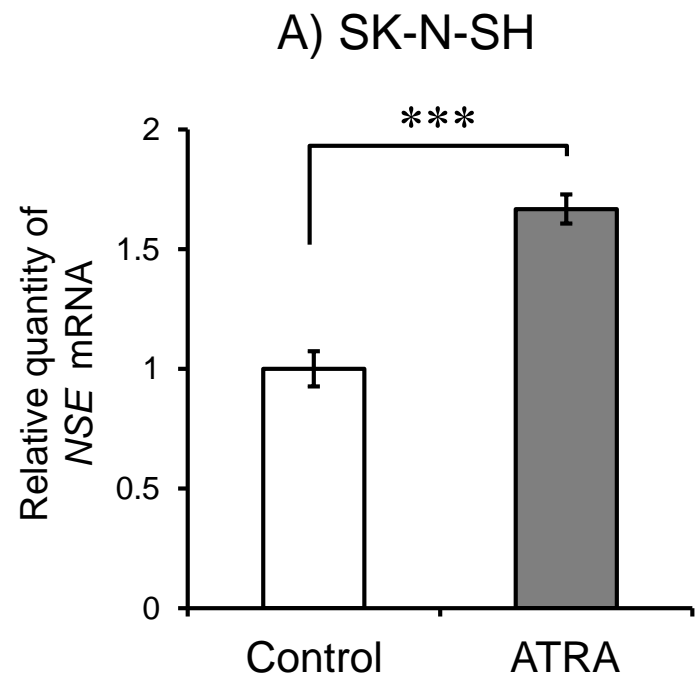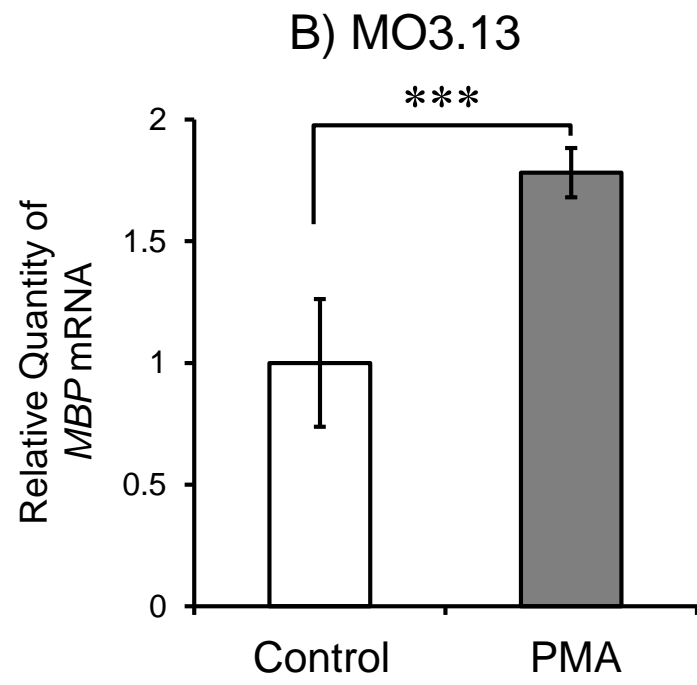

Supplement: Supplementary file 4 [file Presentation_4.PDF]
