# Supplementary material for: Perinatal Asphyxia in Rat Alters Expression of Novel Schizophrenia Risk Genes
Source: Front Mol Neurosci. 2017 Oct 27;10:341. doi: 10.3389/fnmol.2017.00341 (PMC5663725; doi:10.3389/fnmol.2017.00341)
Supplement: Supplementary file 5 [file Data_Sheet_1.docx]

#### SUPPORTING INFORMATION

| **Supplemental table 1.** Gene expression in three brain regions at different time periods following hypoxic insult at birth | | | | | | | | | | |
| --- | --- | --- | --- | --- | --- | --- | --- | --- | --- | --- |
| Gene name | Brain region | C group (vs V group) | | | A group (vs V group) | | | A group (vs C group) | | |
|  |  | P1 | 5W | 12W | P1 | 5W | 12W | P1 | 5W | 12W |
| *Cnnm2* | Pfc | 0.88 ± 0.16 | 1.11 ± 0.11 | 0.82 ± 0.06 | 0.61 ± 0.06 | 0.72 ± 0.05 | 0.86 ± 0.09 | 0.69 ± 0.07 | 0.65 ± 0.04 | 1.03 ± 0.11 |
|  | Str | 0.86 ± 0.14 | 0.99 ± 0.11 | 1.20 ± 0.09 | 0.54 ± 0.06 | 0.90 ± 0.07 | 1.07 ± 0.08 | 0.63 ± 0.07 | 0.90 ± 0.07 | 0.85 ± 0.06 |
|  | Hip | 0.75 ± 0.09 | 1.36 ± 0.18 | 0.83 ± 0.06 | 0.68 ± 0.07 | 1.09 ± 0.06 | 0.97 ± 0.09 | 0.92 ± 0.09 | 0.80 ± 0.04 | 1.18 ± 0.11 |
| *Csmd1* | Pfc | 2.32 ± 0.40 | 1.35 ± 0.10 | 0.97 ± 0.13 | 3.84 ± 1.62 | 0.92 ± 0.09 | 0.70 ± 0.14 | 1.65 ± 0.69 | 0.68 ± 0.07 | 0.75 ± 0.16 |
|  | Str | 1.60 ± 0.23 | 1.19 ± 0.19 | 1.24 ± 0.11 | 1.52 ± 0.14 | 1.10 ± 0.09 | 1.37 ± 0.10 | 0.95 ± 0.09 | 0.92 ± 0.08 | 1.04 ± 0.08 |
|  | Hip | 2.25 ± 0.37 | 1.26 ± 0.24 | 0.83 ± 0.08 | 1.71 ± 0.46 | 1.14 ± 0.11 | 0.98 ± 0.12 | 0.76 ± 0.21 | 0.90 ± 0.09 | 1.19 ± 0.14 |
| *Mmp16* | Pfc | 2.19 ± 0.42 | 1.50 ± 0.13 | 1.03 ± 0.09 | 3.09 ± 1.44 | 0.89 ± 0.12 | 0.93 ± 0.14 | 1.41 ± 0.66 | 0.59 ± 0.08 | 0.95 ± 0.15 |
|  | Str | 1.84 ± 0.37 | 0.99 ± 0.13 | 1.23 ± 0.06 | 1.58 ± 0.21 | 0.96 ± 0.09 | 1.08 ± 0.09 | 0.86 ± 0.11 | 0.97 ± 0.09 | 0.85 ± 0.07 |
|  | Hip | 1.25 ± 0.37 | 1.30 ± 0.18 | 2.00 ± 1.05 | 1.77 ± 0.53 | 1.00 ± 0.08 | 1.00 ± 0.09 | 1.41 ± 0.43 | 0.77 ± 0.06 | 0.50 ± 0.04 |
| qRT-PCR quantification of *Cnnm2*, *Csmd1* and *Mmp16* expression in prefrontal cortex (Pfc), striatum (Str), and hippocampus (Hip) at neonatal (postnatal day 1, P1), adolescence (5-week-old, 5W), and adulthood (12-week-old, 12W). Values are expressed as mean ± SEM. | | | | | | | | | | |

**Supplemental figure 1.** *Cnnm2* expression of different brain regions at different time periods following hypoxic insult at birth. qRT-PCR quantification of *Cnnm2* expression in prefrontal cortex (Pfc) (A) and striatum (Str) (B) at neonatal (postnatal day 1, P1) adolescence (5-week-old, 5W), and adulthood (12-week-old, 12W). Relative *Cnnm2* expression was obtained by normalizing to *Gapdh* from the same cDNA. Results are calculated as a ratio of V group expression. Error bars represent mean ± SEM (V group, *n* = 7; C group, *n* = 6; A group, *n* = 8).

#### Supplemental figure 2. *Csmd1* expression of different brain regions at different time periods following C-section at birth. qRT-PCR quantification of *Csmd1* expression in striatum (Str) (A) and hippocampus (Hip) (B) at neonatal (postnatal day 1, P1) adolescence (5-week-old, 5W), and adulthood (12-week-old, 12W). Relative *Csmd1* expression was obtained by normalizing to *Gapdh* from the same cDNA. Results are expressed as a ratio of V group expression, resulting in a V group ratio of 1. Error bars represent mean ± SEM (V group, *n* = 7; C group, *n* = 6; A group, *n* = 8).

#### Supplemental figure 3. *Mmp16* expression of different brain regions at different time periods following C-section at birth. qRT-PCR quantification of *Mmp16* expression in striatum (Str) at neonatal (postnatal day 1, P1) adolescence (5-week-old, 5W), and adulthood (12-week-old, 12W). Relative *Mmp16* expression was obtained by normalizing to *Gapdh* from the same cDNA. Results are expressed as a ratio of V group expression, resulting in a V group ratio of 1. Error bars represent mean ± SEM (V group, *n* = 7; C group, *n* = 6; A group, *n* = 8).

#### Supplemental figure 4. Differentiation of neuronal and glial cell lines. (A) qRT-PCR quantification of neuronal differentiation maker, NSE, expression of SK-N-SH cells treated with and without ATRA (10 μM) for 72 h. (B) qRT-PCR quantification of oligodendrocytic differentiation maker, MBP, expression of MO3.13 cells treated with and without PMA (100 nM) for 96 h. Error bars represent mean ± SD (*n* = 3–4 per treatment). *** *P* < 0.001 between control cells and cells treated with ATRA (for SK-N-SH cells) or PMA (for MO3.13 cells).
